# A Novel Approach for Detail-Enhanced Exposure Fusion Using Guided Filter

**DOI:** 10.1155/2014/659217

**Published:** 2014-02-09

**Authors:** Harbinder Singh, Vinay Kumar, Sunil Bhooshan

**Affiliations:** ^1^Department of Electronics and Communication Engineering, Baddi University of Emerging Sciences and Technology, Baddi, Solan 173205, India; ^2^Grupo de Procesado Multimedia, Departamento de Teoría de la Señal y Comunicaciones, Universidad Carlo III de Madrid, Leganes, Madrid 28911, Spain; ^3^Department of Electronics and Communication Engineering, Jaypee University of Information Technology, Waknaghat, Solan 173215, India

## Abstract

In this paper we propose a novel detail-enhancing exposure fusion approach using nonlinear translation-variant filter (NTF). With the captured Standard Dynamic Range (SDR) images under different exposure settings, first the fine details are extracted based on guided filter. Next, the base layers (i.e., images obtained from NTF) across all input images are fused using multiresolution pyramid. Exposure, contrast, and saturation measures are considered to generate a mask that guides the fusion process of the base layers. Finally, the fused base layer is combined with the extracted fine details to obtain detail-enhanced fused image. The goal is to preserve details in both very dark and extremely bright regions without High Dynamic Range Image (HDRI) representation and tone mapping step. Moreover, we have demonstrated that the proposed method is also suitable for the multifocus image fusion without introducing artifacts.

## 1. Introduction

**Figure 1:**
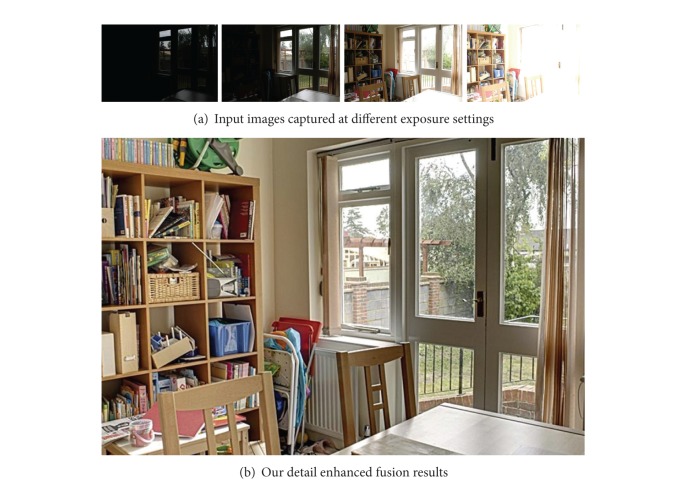
Results of proposed detail-enhanced exposure fusion framework using edge preserving filter.

In single exposure, normal digital camera can collect limited luminance variations from the real world scene, which is termed as low dynamic range (LDR) image. To circumvent this problem, modern digital photography offers the concept of exposure time variation to capture details in very dark or extremely bright regions, which control the amount of light allowed to fall on the sensor. Different LDR images are captured to collect complete luminance variations in rapid successions at different exposure settings known as exposure bracketing. However, each exposure will handle the small portion of the luminance variation in the entire scene. Short exposure can capture details from the bright regions (i.e., highlights) and long exposure can capture details from dark regions (i.e., shadows) (see Figure 1).

In the past decade, two solutions have been proposed to handle large luminance variations present in the natural scenes. The first option is the HDR representation. To date, many HDRI representation [1, 2] techniques have been proposed, which extend dynamic range by compositing differently exposed images of the same scene. HDR images generally encode intensity variations with more than 8-bits and pixel values that are proportional to the true scene radiance, transformed by a nonlinear mapping called the camera response function. Four bytes “hdr” format was developed to encode radiance maps. The second option to encode radiance map is “floating point tiff,” which uses 12 bytes to encode 79 orders of magnitude approximately. Currently used standard display devices have smaller contrast ratio (i.e., 1 : 100) and the contrast ratio of LCD monitors can reach 1 : 400. Recently developed HDR display device prototypes [3] can represent high contrast ratio (i.e., 1 : 25,000), which are still not available in the market for the routine customers. Therefore, the HDR image needs to be tone-mapped first to appear on standard display device. Various local and global tone mapping methods [2] have been proposed to display HDR images on standard display devices. Local light adaption property of human visual system (HVS) is adopted in the local operators to correspond to the visual impression that an observer had when watching the original scene, while the global operators are spatially invariant and are less effective than the local operators.

The recently proposed second option is the “exposure fusion.” The fundamental goal of the exposure fusion is to preserve details in both very dark and extremely bright regions without HDRI representation and tone mapping step. The underlying idea of various exposure fusion approaches [4–7] is based on the utilization of different local measures to generate weight map to preserve details present in the different exposures.

**Figure 2:**
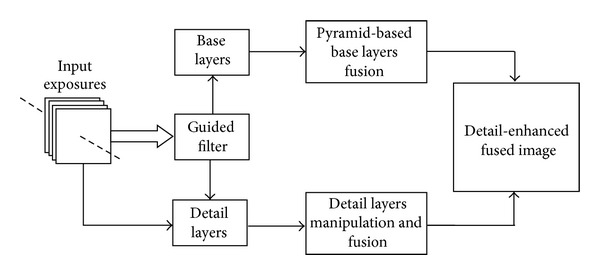
Proposed detail-enhanced exposure fusion framework.

**Figure 3:**
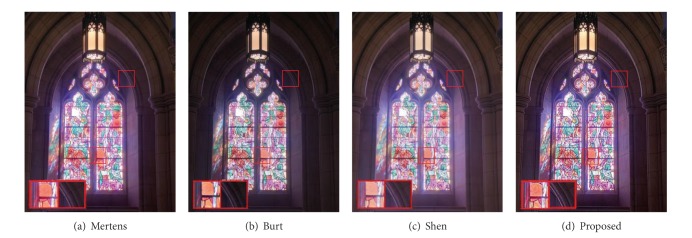
Comparison results to other recent exposure fusion techniques. (a) Mertens et al. [4], (b) Burt and Adelson [13], (c) Shen et al. [7], and (d) results of our new exposure fusion method. Note that our method yields enhanced texture and edge features. Input image sequence is courtesy of Tom Mertens.

**Figure 4:**
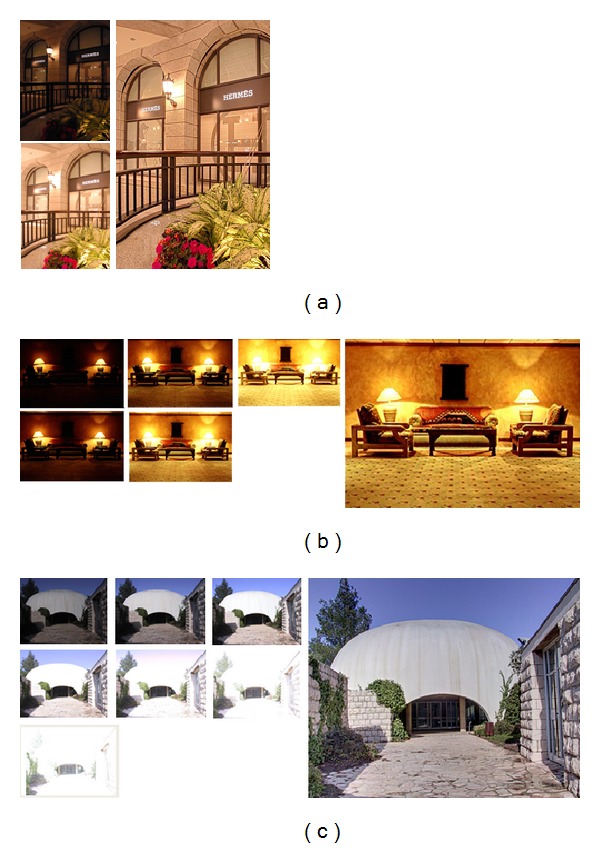
Our results for different multiexposure sequences. (a) Hermes (two input exposures), (b) Chairs (five exposures), and (c) Syn (seven input exposures).

The present work draws inspiration from imaging techniques that combine information from two or more images captured at different exposure settings but with different goals. The block diagrammatic representation of the present detail enhanced framework is shown in Figure 2. We seek to enhance fine details in the fused image by using edge preserving filter [8]. Edge preserving filters have been utilized in several image processing applications such as edge detection [9], image enhancement, and noise reduction [10]. Recently, joint bilateral filter [11] has been proposed which is effective for detecting and reducing large artifacts such as reflections using gradient projections. More recently, anisotropic diffusion [9] has been utilized for detail enhancement in exposure fusion [12], in which texture features are used to control the contribution of pixels from the input exposures. In our approach, the guided filter is preferred over other existing approaches because the gradients present near the edges are preserved accurately. We use guided filter [8] for base layer and detail layer extractions which is more effective for enhancing texture details and reducing gradient reversal artifacts near the strong edges in the fused image. Multiresolution approach is used to fuse computed base layers across all of the input images. The detail layers extracted from input exposures are manipulated and fused separately. The final detail enhanced fused image (see Figures 3 and 4) is obtained by integrating the fused base layer and the fused detail layer. The detailed description of the proposed approach is given in the forthcoming section. It is worth pointing out that our method essentially differs from [4], which aims at enhancing the texture and contrast details in the fused image with a nonlinear edge preserving filter (i.e., the guided filter). Moreover, it is demonstrated that the proposed approach fuses the multifocus images effectively and produces the result of rich visual details.

## 2. Guided Image Filtering for Base Layer Computation

### 2.1. Edge Preserving Guided Filter

In this section, we first describe the ability of the guided filter [8] derived from local linear model to preserve edges, and then show how it avoids gradient reversal artifacts near the strong edges that may appear in fused image after detail layer enhancement. We seek to maintain the shape of strong edges in the fused image that appears due to exposure time variation across input images.

Guided filter was developed by He et al. [8] in 2010 as an alternative to bilateral filter [11]. It is an edge-preserving filter where the filtering output is a local linear model between the guidance *I* and the filter output *q*. The selection of guidance image *I* will depend on the application [11]. In our implementation, an input image *p* and guidance image *I* are identical. The output of the guided filter for a pixel *i* is computed as a weighted averages as follows:

(1) $$\begin{array}{c} q_{i}=\sum_{j}W_{ij}\left(I\right)p_{i}, \end{array}$$

where (*ij*) are pixel indexes and *W*_*ij*_ is the filter kernel that is a function of guidance image *I* and independent of input image *p*. Let *q* be a linear transform of *I* in a window centered at the pixel *k* as follows:

(2) $$\begin{array}{c} q_{i}=a_{k}I_{i}+b_{k},\;\forall i\in \omega_{k}, \end{array}$$

where (*a*_*k*_, *b*_*k*_) are the linear coefficients assumed to be constant in *ω*_*k*_ and calculated in a small square image window of a radius (2*r* + 1)×(2*r* + 1). The local linear model (2) ensures that *q* has an edge (i.e., discontinuities) only if *I* has an edge, because ∇*q* = *a*∇*I*. Here, *a*_*k*_ and *b*_*k*_ are computed within to minimize the following cost function:

(3) $$\begin{array}{c} E\left(a_{k},b_{k}\right)=\sum_{i\in \omega_{k}}\left({\left(a_{k}I_{i}+b_{k}-p_{i}\right)}^{2}+\varepsilon a_{k}^{2}\right), \end{array}$$

where *ε* is the regularization term on linear coefficient a for numerical stability. The significance and relation of *ε* with the bilateral kernel [11] are given in [8]. In our implementation, we use *r* = 2 (i.e., 5 × 5 square window) and *ε* = 0.01.

The linear coefficients used to minimize the cost function in (3) are determined by linear regression [15] as follows:

(4) $$\begin{array}{c} a_{k}=\frac{\left(1/\left|\omega \right|\right)\sum_{i\in \omega_{k}}I_{i}p_{i}-\mu_{k}{\bar{p}}_{k}}{\sigma_{k}^{2}+\varepsilon },\\ b_{k}={\bar{p}}_{k}-a_{k}\mu_{k},\\ {\bar{p}}_{k}=\frac{1}{\left|\omega \right|}\sum_{i\in \omega_{k}}p_{i}, \end{array}$$

where *μ*_*k*_ and *σ*_*k*_^2^ are the mean and variance of *I* in *ω*_*k*_, |*ω*| is the number of pixels in *ω*_*k*_, and *p*_*k*_ is the mean of *p* in *ω*_*k*_.

The linear coefficients *a*_*k*_ and *b*_*k*_ are computed for all patches *ω*_*k*_ in the entire image. However, a pixel *i* is involved in all windows *ω*_*k*_ that contains *i* so the value of *q*_*i*_ in (2) will be different for different windows. So, after taking the average of all the possible value of *q*_*i*_, the filtered output is determined as

(5) qi=1|ω|∑k:i∈ωk(akIi+bk)

(6) =(a−iIi+b−i).

Here, ${\overset{-}{a}}_{i}$ and ${\overset{-}{b}}_{i}$ are computed as

(7) $$\begin{array}{c} {\overset{-}{a}}_{i}=\frac{1}{\left|\omega \right|}\sum_{k\in \omega_{i}}a_{k},\;\;{\overset{-}{b}}_{i}=\frac{1}{\left|\omega \right|}\sum_{k\in \omega_{i}}b_{k}. \end{array}$$

In practice, it is found that ${\overset{-}{a}}_{i}$ and ${\overset{-}{b}}_{i}$ in (7) are varying spatially to preserve strong edges of *I* in *q*, that is, $\nabla q\approx {\overset{-}{a}}_{i}\nabla I$. Therefore, *q*_*i*_ computed in (6) preserves the strongest edges in *I* while smoothing small changes in intensity.

Let *b*_*K*_(*i*′, *j*′) be the base layer computed from (6) (i.e., *b*_*K*_(*i*′, *j*′) = *q*_*i*_ and 1 ≤ *K* ≤ *N*) for *K*th input image denoted by *I*_*K*_(*i*′, *j*′). The detail layer is defined as the difference between the guided filter output and the input image, which is defined as

(8) $$\begin{array}{c} d_{K}\left(i^{\prime },j^{\prime }\right)=I_{K}\left(i^{\prime },j^{\prime }\right)-b_{K}\left(i^{\prime },j^{\prime }\right). \end{array}$$

### 2.2. Computation of Laplacian and Gaussian Pyramid

Researchers have attempted to synthesize and manipulate the features at several spatial resolutions that avoid the introduction of seam and artifacts such as contrast reversal or black halos. In the proposed algorithm, the band-pass [13] components at different resolutions are manipulated based on weight map that determine the pixel value in the reconstructed fused base layer. The pyramid representation expresses an image as a sum of spatially band-passed images while retaining local spatial information in each band. A pyramid is created by lowpass-filtering an image *G*_0_ with a compact two-dimensional filter. The filtered image is then subsampled by removing every other pixel and every other row to obtain a reduced image *G*_1_. This process is repeated to form a Gaussian pyramid *G*_0_, *G*_1_, *G*_2_, *G*_3_,…, *G*_*d*_:

(9) $$\begin{array}{c} G_{l}^{}\left(i,j\right)=\sum_{m}^{}\sum_{n}^{}G_{l-1}^{}\left(2i+m,2j+n\right),\;l=1,\ldots ,d, \end{array}$$

where *l* (0 < *l* < *d*) refers to the number of levels in the pyramid.

Expanding *G*_1_ to the same size as *G*_0_ and subtracting yields the band-passed image *L*_0_. A Laplacian pyramid *L*_0_, *L*_1_, *L*_2_,…, *L*_*d*−1_, can be built containing band-passed images of decreasing size and spatial frequency.

(10) $$\begin{array}{c} L_{l}=G_{l}-G_{l+1},\;l=1,\ldots ,d-1, \end{array}$$

where the expanded image *G*_*l*+1_ is given by

(11) $$\begin{array}{c} G_{l+1}=4\sum_{m}^{}\sum_{n}^{}w\left(m,n\right)\left[G_{l}^{}\left(2i+\frac{m}{2},2j+\frac{n}{2}\right)\right]. \end{array}$$

The original image can be reconstructed from the expanded band-pass images:

(12) $$\begin{array}{c} G_{0}=L_{0}+L_{1}+L_{2}+\cdots +L_{d-1}+G_{d}. \end{array}$$

The Gaussian pyramid contains low-passed versions of the original *G*_0_, at progressively lower spatial frequencies. This effect is clearly seen when the Gaussian pyramid “levels” are expanded to the same size as *G*_0_. The Laplacian pyramid consists of band-passed copies of *G*_0_. Each Laplacian level contains the “edges” of a certain size and spans approximately an octave in spatial frequency.

### 2.3. Base Layer Fusion Based on Multiresolution Pyramid

In our framework, the fused base layer *b*_*f*_(*i*′, *j*′) is computed as the weighted sum of the base layers *b*_1_(*i*′, *j*′), *b*_2_(*i*′, *j*′),…, *b*_*N*_(*i*′, *j*′) obtained across *N* input exposures. We use the pyramid approach proposed by Burt and Adelson [13], which generates Laplacian pyramid of the base layers *L*{*b*_*K*_(*i*′, *j*′)}^*l*^ and Gaussian pyramid of weight map functions *G*{*W*_*K*_(*i*′,*j*′)}^*l*^ estimated from three quality measures (i.e., saturation *S*_*K*_(*i*′, *j*′), contrast *C*_*K*_(*i*′, *j*′), and exposure *E*_*K*_(*i*′, *j*′)). Here, *l* (0 < *l* < *d*) refers to the number of levels in the pyramid and *K* (1 < *K* < *N*) refers to the number of input images. The weight map is computed as the product of these three quality metrics (i.e., *W*_*K*_(*i*′, *j*′) = *S*_*K*_(*i*′, *j*′) · *C*_*K*_(*i*′, *j*′) · *E*_*K*_(*i*′, *j*′)). The *L*{*b*_*K*_(*i*′,*j*′)}^*l*^ multiplied with the corresponding *G*{*W*_*K*_(*i*′,*j*′)}^*l*^ and summing over *K* yield modified Laplacian pyramid *L* ^*l*^(*i*′, *j*′) as follows:

(13) $$\begin{array}{c} L^{l}\left(i^{\prime },j^{\prime }\right)=\sum_{K=1}^{N}L\left\{b_{K}^{l}\left(i^{\prime },j^{\prime }\right)\right\}G\left\{W_{K}^{l}\left(i^{\prime },j^{\prime }\right)\right\}. \end{array}$$

The *b*_*f*_(*i*′, *j*′) that contains well exposed pixels is reconstructed by expanding each level and then summing all the levels of the Laplacian pyramid:

(14) $$\begin{array}{c} b_{f}\left(i^{\prime },j^{\prime }\right)=\sum_{l=0}^{d}L^{l}\left(i^{\prime },j^{\prime }\right). \end{array}$$

### 2.4. Detail Layer Fusion and Manipulation

The detail layers computed in (8) across all the input exposures are linearly combined to produce fused detail layer *d*_*f*_(*i*′, *j*′) that yields combined texture information as follows:

(15) $$\begin{array}{c} d_{f}\left(i^{\prime },j^{\prime }\right)=\frac{\sum_{K=0}^{N}\gamma f_{K}\left(d_{K}\left(i^{\prime },j^{\prime }\right)\right)}{N}, \end{array}$$

where *γ* is the user defined parameter to control amplification of texture details (typically set to 5) and *f*_*K*_(·) is the nonlinear function to achieve detail enhancement while reducing noise and artifacts near strong edges due to overenhancement. We follow the approach of [10] to reduce noise across all detail layers. The nonlinear function *f*_*K*_(·) is defined as

(16) $$\begin{array}{c} f_{K}\left(i^{\prime },j^{\prime }\right)=\tau {\left(d_{K}\left(i^{\prime },j^{\prime }\right)\right)}^{\alpha }+\left(1-\tau \right)d_{K}\left(i^{\prime },j^{\prime }\right), \end{array}$$

where *τ* is a smooth step function equal to 0 if *d*_*K*_(*i*′, *j*′) is less than 1% of the maximum intensity, 1 if it is more than 2%, with a smooth transition in between, and the parameter *α* is used to control contrast in the detail layers. We have found that *α* = 0.2 is a good default setting for all experiments.

Finally, the detail enhanced fused image *g*(*i*′, *j*′) is easily computed by simply adding up the fused base layer *b*_*f*_(*i*′, *j*′) computed in (14) and the manipulated fused detail layer *d*_*f*_(*i*′, *j*′) in (15) as follows:

(17) $$\begin{array}{c} g\left(i^{\prime },j^{\prime }\right)=d_{f}\left(i^{\prime },j^{\prime }\right)+b_{f}\left(i^{\prime },j^{\prime }\right). \end{array}$$

## 3. Experimental Results and Analysis

### 3.1. Comparison with Other Exposure Fusion Methods

Figures 1, 3, and 4 depict examples of fused images from the multiexposure images. It is noticed that the proposed approach enhances texture details while preventing halos near strong edges. As shown in Figure 1(b), the details from all of the input images are perfectly combined and none of the four input exposures (see Figure 1(a)) reveals fine textures on the chair that are present in the fused image. In Figures 3(a)–3(d), we compare our results to the recently proposed approaches. Figures 3(a) and 3(b) show the fusion results using the multiresolution pyramid based approach. The result of Mertens et al. [4] (see Figure 3(a)) appears blurry and loses texture details while in our results (see Figure 3(d)) the wall texture and painting on the window glass are emphasized which are difficult to be visible in Figure 3(a). Clearly, this is suboptimal as it removes Pixel-to-pixel correlations by subtracting a low-pass filtered copy of the image from the image itself to generate a Laplacian pyramid and the result is a texture and edge details reduction in the fused image.  Figure 3(b) shows the results using pyramid approach [13] which reveals many details but losses contrast and color information. Generalized random walks based exposure fusion is shown in Figure 3(c) which depicts less texture and color details in brightly illuminated regions (i.e., lamp and window glass). Note that Figure 3(d) retains colors, sharp edges, and details while also maintaining an overall reduction in high frequency artifacts near strong edges.

Figure 4 shows our results for different image sequences captured at variable exposure settings (see Figure 4(a), Hermes; Figure 4(b), Chairs; and Figure 4(c), Syn (input images are courtesy of Jacques Joffre and Shree Nayar)). Note that, the strong edges and fine texture details are accurately preserved in the fused image without introducing halo artifacts. The halo artifacts will stand out if the detail layer undergoes a substantial boost.

**Figure 5:**
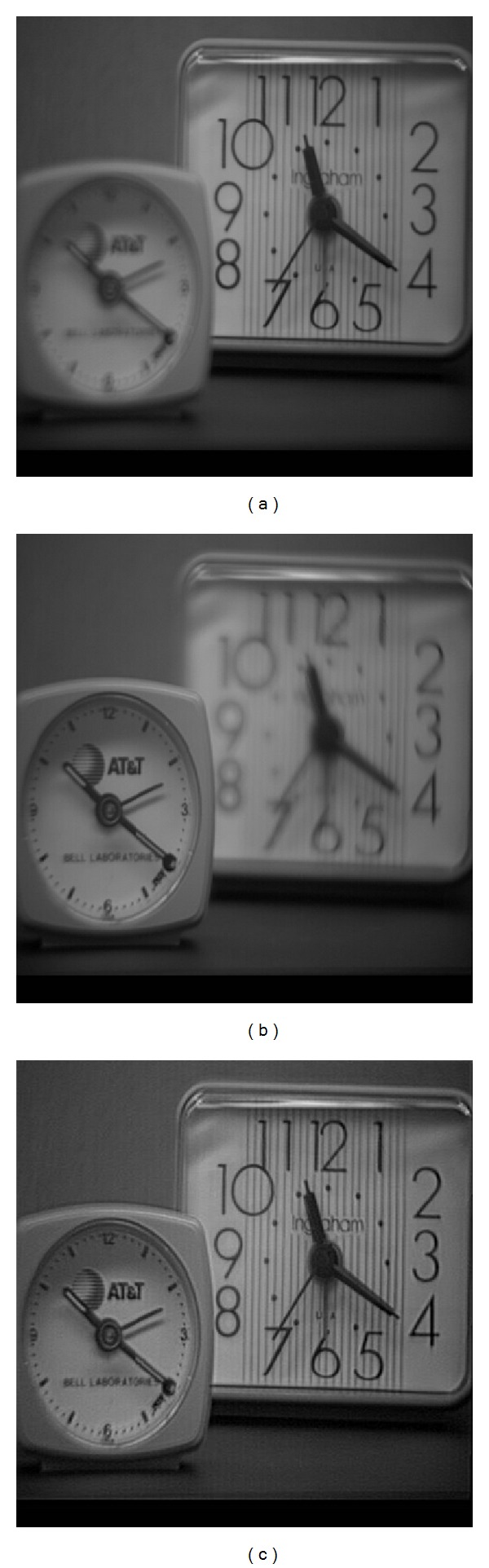
((a), (b)) Two partially focused images (focused on different targets), (c) image generated by the proposed approach, which illustrates that the fused image extracts more information from the original images. Input sequence is courtesy of Adu and Wang.

Moreover, in Figure 5, it is demonstrated that the proposed method is also suitable for multifocus image fusion to yield rich contrast. As illustrated in Figure 5(c), the edges and textures are relatively better than those of input images. Because our approach excludes fine textures from the base layers, we can significantly preserve and enhance fine details separately. However, multiresolution pyramid approach can be accurately used for retaining strong edges and texture details enhancement in multifocus image fusion problem.

### 3.2. Implementation and Comparison of Various Classic Edge-Preserving Filters

**Figure 6:**
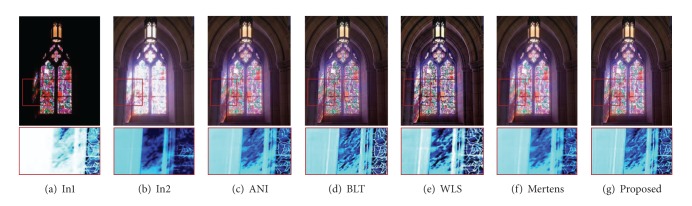
Color-coded map comparison (dark blue color indicates overexposed region and pure white indicates underexposed region). Comparison results to other classic edge preserving and exposure fusion techniques. (a) Source exposure 1; (b) source exposure 2; (c) anisotropic filter [9]; (d) bilateral filter [11]; (e) weighted least square filter [14]; (f) Mertens [4]; (g) results of our new exposure fusion method based on guided filter. Note that our method yields enhanced texture and edge features. Input image sequence courtesy of Tom Mertens.

Figures 6(a) and 6(b) depict the color-coded maps of underexposed and overexposed images, respectively. Dark blue color indicates overexposed region and pure white color indicates underexposed region. Figures 6(c)–6(f) illustrate the comparisons of color-coded maps of three edge preserving filters based detail enhancement and the results obtained by Mertens et al. [4] on the Cathedral sequence. We accepted the default parameter settings suggested by the different edge preserving filters [9, 11, 14]. Figures 6(c) and 6(f) show, respectively, the fusion results using the anisotropic diffusion [9] based approach and the multiresolution pyramid based exposure fusion approach [4], which are both clearly close to the results obtained using guided filter (see Figure 6(g)), but overall, they yield less texture and edge details. The texture detail enhancement using bilateral filter [11] and weighted least square filter [14] shown in Figures 6(d) and 6(e), respectively, depicts overenhancement near strong edges and less color details. As shown in the close-up view in Figure 6(g), the proposed method based on guided filter can enhance the image texture details while preserving the strong edges without over enhancement.

### 3.3. Analysis of Free Parameters and Fusion Performance Metrics

**Figure 7:**
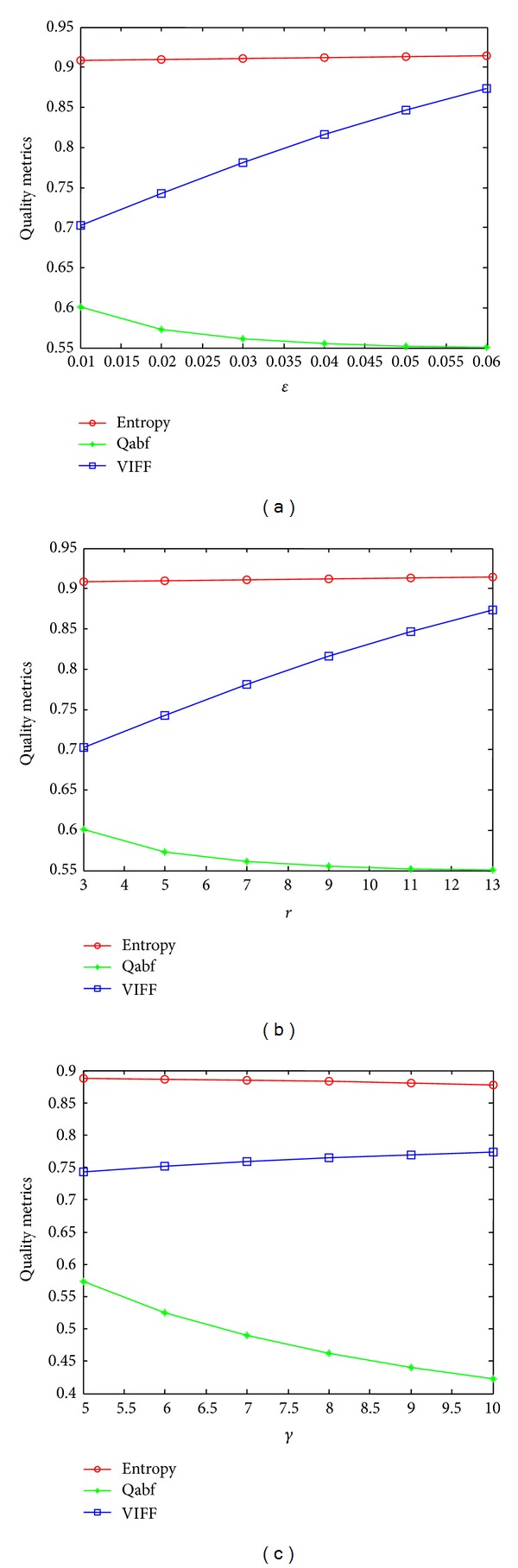
Analysis of different free parameters used in the algorithm. Maximum quality score and entropy are only observed when *ε* = 0.01, *γ* = 5, and *r* = 2 (which are set as default parameters). It is observed that VIFF increases as *ε*, *γ*, and *r* increases but the larger values are responsible for overenhancement. (a) Effectiveness of *ε* on metrics, (b) effectiveness of *r* on metrics, (c) effectiveness of *γ* on metrics.

To analyze the effect of epsilon, gamma, and window size on quality score (Qabf) [16], entropy, and visual information fidelity for fusion (VIFF) [17], we have illustrated three plots (see Figures 7(a)–7(c), resp.) for input image sequence of “Cathedral.” To assess the effect of epsilon, gamma, and window size on fusion performance, the Qabf, entropy, and VIFF were adopted in all experiments executed on a PC with 2.2 GHz i5 processor and 2 GB of RAM. VIFF [17] first decomposes the source and fused images into blocks. Then, VIFF utilizes the models in VIF (GSM model, distortion model, and HVS model) to capture visual information from the two source-fused pairs. With the help of an effective visual information index, VIFF measures the effective visual information of the fusion in all blocks in each subband. Finally, the assessment result is calculated by integrating all the information in each subband. Qabf [16] evaluates the amount of edge information transferred from input images to the fused image. A Sobel operator is applied to yield the edge strength and orientation information for each pixel.

**Figure 8:**
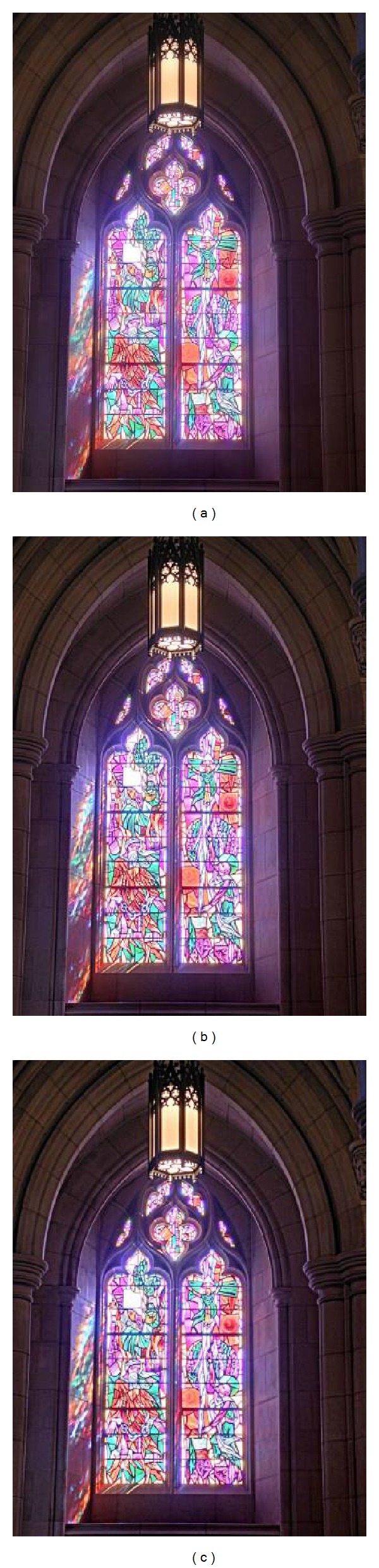
Visual inspection: the effect of free parameter *r* on detail enhancement. We have found that *r* = 2 is sufficient for fine details extraction and gives better results for most cases. Higher value of *r* brings in artifacts near strong edges. (a) *r* = 1; (b) *r* = 3; and (c) *r* = 6.

First, to analyze the effect of *ε* on Qabf, entropy, and VIFF, the square window parameter (*r*) and texture amplification parameter (*γ*) were set to 2 and 5, respectively. As shown in Figure 7(a), the quality score and entropy decreases as *ε* increases and VIFF increases as *ε* increases. It should be noticed in Figure 7(b) that the VIFF and entropy increase as *r* increases and Qabf decreases as *r* increases. It is preferred to have a small filter size (*r*) to reduce computational time. In the analysis of *r*, the other parameters are set to *ε* = 0.01 and *γ* = 5. The visual inspection of effect of *r* on “Cathedral” sequence is depicted in Figure 8. It can easily be noticed (see Figures 8(a)–8(c)) that as *r* increases, the strong edges and textures get overenhanced and therefore leads to artifacts. To analyze the influence of *γ*, it should be noticed that entropy and Qabf decrease as *γ* increases and VIFF increases as *γ* increases. In order to obtain optimal detail enhancement and low computational time, we have concluded that the best results were obtained with *ε* = 0.01, *γ* = 5, and *r* = 2, which yield reasonably good results for all cases.

## 4. Conclusions

We proposed a method to construct a detail enhanced image from a set of multiexposure images by using a multiresolution decomposition technique. When compared with the existing techniques which use multiresolution and single resolution analysis for exposure fusion, the current proposed method performs better in terms of enhancement of texture details in the fused image. The framework is inspired by the edge-preserving property of guided filter that has better response near strong edges. The two layer decomposition based on guided filter is used to extract fine textures for detail enhancement. Moreover, we have demonstrated that the present method can also be applied to fuse multifocus images (i.e., images focused on different targets). More importantly, the information in the resultant image can be controlled with the help of the proposed free parameters.
